# Is Overweight in Stunted Preschool Children in Cameroon Related to Reductions in Fat Oxidation, Resting Energy Expenditure and Physical Activity?

**DOI:** 10.1371/journal.pone.0039007

**Published:** 2012-06-11

**Authors:** Rihlat Said-Mohamed, Jonathan Y. Bernard, Anne-Christine Ndzana, Patrick Pasquet

**Affiliations:** 1 UMR 7206 Eco-anthropologie et Ethnobiologie, Centre National de la Recherche Scientifique, Muséum national d’Histoire naturelle, Paris Diderot University, Paris, France; 2 Centre for Food and Nutrition Research, Institute of Medical Research and Medicinal Plant Studies, Yaoundé, Cameroon; Pennington Biomed Research Center, United States of America

## Abstract

**Background:**

Recent studies suggest that early modifications in metabolic pathways and behaviour, leading to energy conservation and reduced linear growth, could represent adaptations to nutritional constraints during foetal life and infancy. Impaired fat oxidation, low resting energy expenditure and reduced physical activity, resulting from these adaptations, could facilitate fat storage and development of overweight in growth-retarded children that consume more energy-dense food. This study aims at assessing whether: (1) dual-burden preschool children (simultaneously stunted and overweight) of Yaounde (Cameroon) have low birth-weight (indicator of foetal undernutrition) and reductions in fat oxidation, resting energy expenditure (REE) and physical activity, (2) fat oxidation, REE and physical activity are associated with foetal growth.

**Methodology/Principal Findings:**

162 children (24–72 months) were considered: 22 stunted-overweight (SO), 40 stunted (S), 41 overweight (O), and 59 non stunted-non overweight (NSNO). Nutritional status and body composition were assessed using anthropometry and multifrequency bioimpedance analysis. Fasting respiratory quotient (RQ) and REE were measured by indirect calorimetry. Physical activity was determined using accelerometers, food questionnaires were used for diet assessment and birth-weight was noted. Mean RQs and REE (weight adjusted) did not differ between stunted children (SO and S) and non-stunted children (O and NSNO). SO and S children spent more time in sedentary activities than O children (p* = *0.01 and p* = *0.02, respectively) and less time in moderate-to-vigorous activities than NSNO children (p* = *0.05 and p* = *0.04, respectively). SO children’s diet was less diverse (p* = *0.01) with less animal products (p* = *0.006). Multiple linear regressions model revealed that birth-weight is predictive of RQ (β = 0.237, p<0.01, R^2^ = 0.08).

**Conclusions/Significance:**

This study showed that growth retardation in stunted-overweight children could be associated with postnatal nutritional deficiencies. Overweight in stunted children could be associated with reduced physical activity in the context of nutrition transition. High birth-weight was a predictor of reduced lipid oxidation, a risk factor of fat deposition.

## Introduction

Nutrition transition, *i.e*. changes in food consumption habits and lifestyle towards a nutritional energy densification and a decreased physical activity, has been reported in several developing countries [1]. These factors are associated with an increased prevalence of obesity and chronic disease in people from all age groups living in economically developed areas, while undernutrition remains a public health concern in less developed areas [2]. This simultaneous occurrence of under- and overnutrition within a population is known as the double burden of malnutrition [3]. Moreover, epidemiological studies found high prevalences of obese mothers-stunted children pairs (the dual burden household) and the coexistence of overweight and stunting within the same person (the dual burden at an individual level) [4].

In fact, during the last two decades, epidemiological studies have revealed that children and adolescents living in countries undergoing nutrition transition can be simultaneously growth retarded and overweight [5]–[11]. An ecological study conducted in Mexico found that maternal factors (short maternal stature, lower maternal age, level of education and perceived social status) and household properties (living in large household, a low socio-economic status) are independent factors associated with the coexistence of overweight and stunting in preschool children [10]. In Yaoundé, the capital city of Cameroon, we previously found that a low socio-economic status, a low level of maternal education and maternal under-evaluation of children’s weight are independent risk factors for these children to be simultaneaously stunted and overweight [11].

Most of the studies on individual dual burden have reported a relatively higher risk for stunted children to become overweight. Meanwhile, food restriction during the early stages of life, among the factors involved in growth delay, can result in predisposition to fat gain in later life [1], [12]–[14]. Varela-Silva et al. [15] proposed an etiological model of stunting concomitant to overweight, centered around the “thrifty phenotype hypothesis” [16]. This hypothesis predicts that maternal constraints during intrauterine and neonatal life stages induce modifications in glucose metabolism, growth patterns and functioning of vascular tissues. Accordingly, these modifications favor survival in a nutritionally restrictive environment, but become detrimental once this environment changes, by inducing a metabolic syndrome that promotes the development of overweight and subsequent chronic diseases. It has been suggested that foetal and neonatal undernutrition may induce growth retardation and *energy sparing mechanisms*, such as impaired fat oxidation, decreased resting energy expenditure and low physical activity [15], [17]–[21]. Therefore, by promoting fat storage they may result in overweight in stunted children when the energy supply has increased. This model has never been studied directly in children which are simultaneously stunted and overweight.

In Yaoundé, stunted preschool children have a relatively higher risk (1.6 times) to become overweight [22], this nutritional status being associated with poverty and maternal factors [11]. In this context, the main objective of this study was to assess if the etiological model presented above could explain the coexistence of stunting and overweight in children living in Yaoundé, by comparing children with contrasted growth and nutritional status. Firstly, nutritional constraints during children’s foetal life, in particular in stunted-overweight children, were investigated using low birth-weight as an indicator of reduced foetal growth. Then, the hypothesis was tested that stunted-overweight children have reduced fat oxidation, low resting energy expenditure and less physical activity level. Finally, the relationships between early life growth (using birth-weight as an indicator) and fat oxidation, resting energy expenditure and physical activity were evaluated.

## Methods

### Screening Study

From December 2008 to February 2009, we made a representative nutritional epidemiological study that included 1895 children (24–72 months) randomly recruited from kindergartens and households of Yaoundé.

After having carefully verified the child’s age, anthropometrical measurements were taken by two trained fieldworkers using standardised procedures [23]. Children’s body height was measured to the nearest millimiter using a portable stadiometer (Siber Hegner, Switzerland). Body weight was measured to the nearest 100 gram with a digital scale.

Nutritional indexes, body mass index (BMI) for age percentile, weight-for-age z-score and height-for-age z-score and percentile were calculated with the Epi info 2000 software, using the sex-specific CDC (Centers for Disease Control and Prevention) reference curves (2000). Overweight was defined as body mass index-for-age (BMI) equal or higher than the 85^th^ percentile. Stunting was defined as height-for-age equal to or lower than the 3^rd^ percentile.

### Subjects

In March 2009, children from the above screening study were randomly pre-included in the study according to their nutritional status. A child was definitively included in the present study after a paediatrician had verified the absence of acute health problems (malaria, infections, chronic respiratory problems) and a 12 hour fasting period. Children with malaria or infections were given medical treatments according to the Research Centre in Food and Nutrition of Yaoundé (RCFN) protocol. 162 children (24 to 72 months) were finally included in the study:

40 stunted children (S): height-for-age (HAC) equal to or below the 3rd percentile, BMI below the 85th percentile and weight-for-height (WHZ) above −2 z-score
41 overweight children (O): HAC above the 3rd percentile and BMI equal or above the 85th percentile
22 stunted and overweight children (SO): HAC equal or below the 3rd percentile and BMI equal or above 85th percentile
59 non-stunted and non overweight (NSNO) children: HAC above the 3^rd^ percentile, BMI below the 85^th^ percentile and WHZ above −2 z-score.

In practice, pre-included children (3 per day) and their parents (or caregivers) were brought to the RCFN at 7∶00 am. After the paediatrician’s examination, the subject’s birth-weight and pre-maturity status were noted from children’s vaccination booklets (Expanded Program on Immunization). For each subject, body composition, metabolic measurements and interviewer-administered food consumption questionnaires were conducted on the same day at the RCFN.

### Body Composition

Body composition was assessed by multifrequency bioelectrical impedance analysis, BIA (at 5, 50, 100, 200 kHz), using a Bodystat Quadscan 4000 (Bodystat, Douglas, United Kingdom). The subject’s body weight, height and waist circumference were measured and entered into the device together with gender and age. The subject was lain in a supine position, electrodes were placed following manufacturer’s instruction and then measurements were performed. The resistance value at 50 kHz (R_50_) was read to the nearest 0.1Ω from a digital display and recorded. Total body water (TBW) was approximated by an impedance index, the ratio of the square of height (H) to Z_200_ (H^2^/Z_200_) [24]. Throughout the study, this ratio was used as an indicator of the children’s leanness, rather than calculating the TBW and the fat free mass from existing BIA equations of prediction. Although the Bodystat Quadscan 4000 can be used at all ages, the prediction equations included are not suitable for children under 6 years. In fact, few predictive equations are available for preschool children and moreover they are virtually non-existent for African children of this age [25], [26]. In addition, specific BIA equations available in the literature were constructed from data on healthy children. Recent studies [27]–[28] advice against their use for children who have undergone recent changes in their body composition, which is the case for both overweight children and growth retarded children. Indeed, stunting has been associated with less expansion of intracellular body water compartment than expected during normal growth of cell mass while fat mass is preserved [29]. Moreover, in obese children, fat free mass was characterised by greater hydration and reduced density [30].

### Indirect Calorimetry

Indirect calorimetry was used to assess the substrate utilization, by calculating children’s respiratory quotient, and resting energy expenditure. Respiratory quotient (RQ = carbon dioxide production/oxygen consumption) and resting energy expenditure (REE) were measured in fasting condition with the QUARK RMR respirometer (Cosmed, Rome, Italy) [31].To ensure that children were fasting, mothers/caregivers were phone called the day before the measurements to remind them to give the dinner to their child before 9∶00 pm and not to feed him until the following morning when our team came to bring them at the RCFN. The next day morning, when we met them, mothers/caregivers ensured that they followed the instructions and that their child was fasting. When it was not the case, we agreed for an appointment another day. At the RCFN, a breakfast was given to children once the measurements were done. Every morning, the calorimeter was calibrated with a standard gas mixture (96% O_2_ and 4% CO_2_). Measurements were taken in thermoneutral conditions with the child lain on a medical table. After a certain period to allow subjects to adjust to wearing the canopy, the measures were recorded during 20 minutes while ensuring that the child remained calm without falling asleep. The monitor measures oxygen consumption (VO_2_, in ml/min) and carbon dioxide production (VCO_2_, in ml/min) and calculates RQ and REE (kJ/day) using the Weir formula [32]. The data corresponding to a stable state [33] were used for the analysis.

### Food Habits

Since no routine methods exist to accurately quantify food intake in these working conditions, for each child, we calculated a dietary diversity score (DDS) based on their 24 hours dietary recall as proposed by Savy and Colleagues [34]. Mothers reported all dishes and beverages consumed by their child the day before experimental measurements. The foods items were grouped into seven categories, according to Arimond and Ruel [35]: 1.starchy staples, 2.legumes, 3.dairy, 4.meat/poultry/fish or eggs, 5.vitamin A rich fruits and vegetables, 6.other fruit and vegetable foods or fruit juice, 7.foods made with oil, fat or butter. The DDS represents the sum of the occurrences of the food groups. The percentage of children who consumed at least one item included in the food group was also calculated for each subject category.

### Physical Activity

Physical activity was measured with an uniaxial ActiGraph GT1M accelerometer (Actigraph Pensacola, FL, USA). Subjects wore the monitor on their right hip (anterior to the iliac crest) with an elastic belt and an adjustable buckle. Measurements lasted six consecutive days and five consecutive nights including the week-end. Counts were registered for an epoch of 60 seconds. Monitors were recovered at the subjects’ home or schools. Date and time were noted and data were subsequently transferred into a computer. Monitors activation and data acquisition were performed with the Actilife Lifestyle Monitoring System software (version 3.2.11). For each subject, the data were reduced. Atypical activities, as reported by the parents or caregivers, were excluded (e.g. when the subjects were ill or when they missed school, for those who were going to school ). Outliers and suplementary data (recorded over 144 hours of programmed measurement) were removed. Periods when the monitor was not worn - which had to be excluded – had to be distinguished from periods of inactivity, since both result in 0 counts/minute. In the literature, the definition of the non-wearing period varies from 10 minutes without activity to 180 minutes [36]. In this study, periods longer than 120 minutes without activity, mean time between two non-zero activities during the night in our subjects, were excluded.

The cut-off points of Puyeau and colleagues [37] were used to discriminate between the activity levels. These four thresholds are: sedentary (≤800 counts/min.), light (≤3200 counts/min.), moderate (≤8200 counts/min.) and vigourous (>8200 counts/min.). We combined moderate and vigorous activities, as recommended for an epoch of 1 minute [36], [38]. For each subjects, the activity counts per minute and per day and the time spent on each activity level were calculated.

### Statistics

All data analyses were carried out with STATISTICA software (version 6.0, Statsoft, Tulsa, OK, USA). Group comparisons were performed using analysis of variance and analysis of covariance. Statistics are presented as mean ± standard deviation. When significant differences were found, post hoc pair-wise comparisons were conducted to determine to which groups they referred, using Tukey’s correction for multiple testing. Multiple regression analysis models were performed to evaluate the relationships between RQ, REE or physical activity (dependant variables) and birth-weight, age and gender (independent variables). Gender was entered as a dummy variable. Spearman correlations analyses were performed to assess the relationship between low and high birth-weight and physical activity.

### Ethics

Research authorization was obtained from the Ministry of Scientific Research and Technological Innovation of Cameroon. The procedures followed were approved by the Institutional Ethic Committee of the Institute of Medical Research and Medicinal Plant Studies of Cameroon. The approval of each District Inspection was obtained for research in kindergartens and directors of the schools had to give their final authorization before study initiation. A written informed consent was obtained from the subjects’ parents.

## Results

There were no significant differences between study groups in either their sex-ratio or age (Table 1). However, there were significantly more boys than girls in the SO group.

**Table 1:** Characteristics of the four groups of subjects.

	Stunted		Stunted-Overweight		Overweight		Non-stunted-Non overweight		p^4^
	n	Means±SD	n	Means±SD	n	Means±SD	n	Means±SD	
Girls	19		5		20		28		ns
Boys	21		17		21		31		
Age (months)	40	49.7±15.6	22	49.5±14.2	41	46.2±11.8	59	50.7±13.1	ns
Birth-weight (kg)^2^	28	3.1±0.66	17	3.4±0.66	35	3.5±0.66	52	3.4±0.66	0.064
***Nutritional status***									
Height (cm)	40	91.5±8.4	22	91.0±8.8	41	99.7±7.4	59	101.8±7.6	***
Weight (kg)	40	13.2±2.3	22	14.9±2.5	41	17.6±2.5	59	16.2±2.4	***
BMI	40	15.7±0.9	22	17.9±1.1	41	17.6±0.7	59	15.6±1.0	***
HAZ^1^	40	−2.53±0.68	22	−2.71±1.09	41	−0.13±0.74	59	−0.25±0.88	***
WAZ^1^	40	−1.99±0.86	22	−0.92±0.85	41	0.82±0.55	59	−0.22±0.80	***
z-BMI	40	0.03±0.70	22	1.53±0.49	41	1.39±0.33	59	−0.01±0.81	***
***Body composition***									
R_50_ (Ω)^1^	40	831±93	22	723±87	41	719±75	59	783±90	***
Z_200_ 1	40	11.3±2.9	22	12.7±3.2	41	15.4±3.3	59	14.7±3.0	***
H^2^/Z_200_ 3	40	13.6±1.7	22	13.2±1.3	41	13.3±1.8	59	13.9±1.3	*

1 HAZ: height-for-age z-score; WAZ: weight-for-age z-score; R_50_: resistance at 50 kHz; Z_200_: Impedance at 200 Hz.

2 Sex and prematurity adjusted.

3 Age, gender and weight adjusted.

4* *P<*0.05, ***P<0.001, ns: non significant.

Regarding body composition, the impedance index H^2^/Z_200_, a proxy of total body water concentration, is significantly different between the four groups of children (for all pair-wise comparisons p<0.001, except for O *vs* NSNO p<0.05) (Table 1). NSNO children have the highest mean value of H^2^/Z_200_, followed by S, O and SO children having the lowest values. On average, the H^2^/Z_200_ ratio is higher in girls than boys (14.0±1.3 *vs* 13.4±1.3 kg, respectively p<0.01).

**Figure 1:**
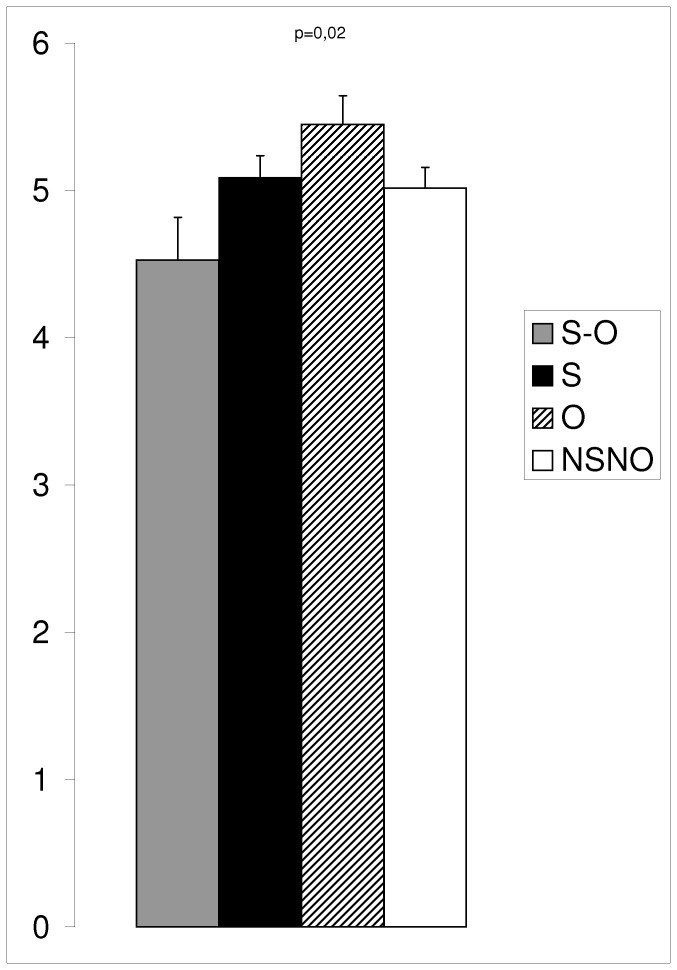
Mean dietary diversity score (DDS) for each group of children. S-O: stunted-overweight children (n = 19), S: Stunted children (n = 35), O: Overweight children (n = 38), NSNO: Non-stunted-Non Overweight children (n = 59). p-value corresponds to the analysis of variance between the four groups. Error bars represent standard error of the mean. S-O children have a significantly less diversified diet than O children (p = 0.01, post hoc pair-wise comparison).

**Figure 2:**
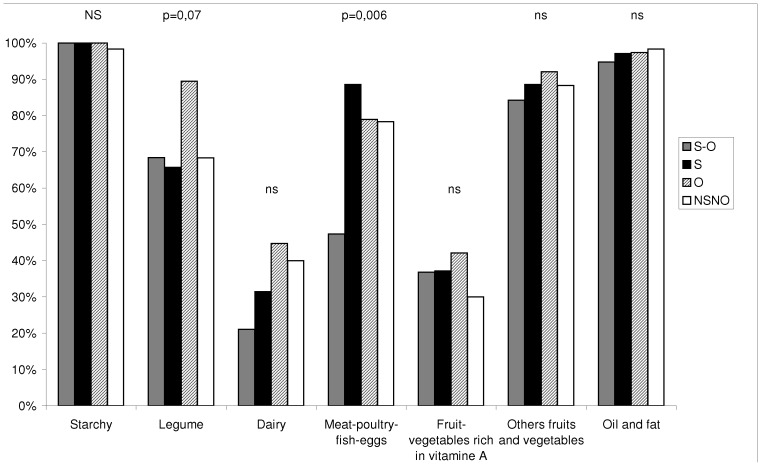
Mean percentage, per group, of children who consumed one item of the food group. S-O: stunted-overweight children (n = 18), S: Stunted children (n = 34), O: Overweight children (n = 37), NSNO: Non-stunted-Non Overweight children (n = 59). p-values correspond to the Pearson’s chi-squared test; ns =  non significant. Relationships do exist between the consumption of “meat-poultry-fish-eggs” and the different nutritional status of children (p = 0.006): S-O children are less likely to consume these items than NSNO (p = 0.01), O (p = 0.01) and S (p = 0.001) children.

We documented substantial differences in the children’s diet (Figure 1 and 2). The mean dietary diversity score for SO children was significantly lower than the one for O children (p* = *0.01 in post hoc pair-wise comparison) (Figure 1). Figure 2 shows within each food group, the mean percentage of children of each group of children, who consumed at least one item included in the given food group. The four groups of children had a similar qualitative pattern of food consumption (Figure 2). Nevertheless, there are significantly less SO children who consumed meats, poultry, fish and eggs (*vs* S p* = *0.001, *vs* O p* = *0.01, *vs* NSNO p* = *0.01). Stunted children (as a group) are fewer to consume dairy products than non-stunted children (as a group) (p* = *0.05).

No group differences were found concerning fasting RQ (Table 2).

**Table 2:** Respiratory quotient (RQ) and resting energy expenditure (REE) of subjects.

	Stunted		Stunted-Overweight		Overweight		Non-stunted-Non overweight		p^3^
	n	Means±SD	n	Means±SD	n	Means±SD	n	Means±SD	
RQ^1^	38	0.786±0.038	22	0.795±0.037	41	0.796±0.038	59	0.786±0.038	ns
REE (kJ.d^−1^)^2^	38	2 669±483	22	2 888±486	41	3 423±486	59	3 051±484	***
REE (kJ.d^−1^.kg^−1^ of body weight) 2	38	202.3±27.3	22	198.0±27.6	41	189.8±27.5	59	190.8±27.4	ns
REE (kJ.d^−1^ Body weight adjusted) 2	38	3 076±542	22	3 024±430	41	2 973±579	59	2 958±431	ns

1 Age adjusted.

2 Age and sex adjusted.

3*** *P<*0.001, ns: non significant.

REE (kJ.d^−1^) was significantly different between the four groups (Table 2). O children had a significantly higher REE (kJ.d^−1^) than S, SO and NSNO children (p* = *8.10^−6^, p* = *0.01, p* = *0.03, respectively). NSNO had a higher REE (kJ.d^−1^) than S children (p* = *0.0005, post hoc pair-wise comparison). Finally, S and SO children did not differ in REE (kJ.d^−1^) (p = 0.14, post hoc pair-wise comparison).

However, after adjustment for weight and when REE is expressed per kilogram of weight, REE was not different between the four groups. Nevertheless, by contrast, stunted children (S+SO) had a higher REE per unit of body weight than non-stunted children (O+NSNO) (p* = *0.03).

Boys had a higher REE than girls (3161±649 kJ.d^−1^ and 2860±576 kJ.d^−1^, respectively, p* = *0.002) even after adjustment for weight (p* = *0.003) or expressed per kilogramme of weight (p* = *0.008).

Overall, children spent most time doing minimal and sedentary activities (Table 3). In the multiple regression model (Table 4), age was positively associated with the total activity count. Table 3 shows that O children were globally more active than S children (p*<*0.001). Indeed, S children performed less light activities (p*<*0.001) than O children. S and SO spent more time in sedentary activities than O children (p* = *0.01 for each group in post hoc pair-wise comparison). S and SO children spend less time doing moderate-to-vigorous activities than NSNO children (p* = *0.03 and p* = *0.05, respectively in post hoc pair-wise comparison).

**Table 3:** Physical activity for the four groups of children (gender, age and “being at school” adjusted).

	Stunted		Stunted-Overweight		Overweight		Non-stunted-Non overweight		p^2^
	n	Means±SD	n	Means±SD	n	Means±SD	n	Means±SD	
Total activity counts (Counts.min^−1^.day^−1^)	38	304.5±84.2	18	319.4±83.8	38	387.1±83.6	39	343.1±84.4	***
***Minutes per day spent in*** **:**									
Minimal (C = 0)^1^	38	692.7±60.1	18	663.9±59.8	38	663.1±59.7	39	676.8±60.2	ns
Sedentary (0<C≤800)^1^	38	552.9±50.7	18	564.4±50.4	38	512.9±50.4	39	541.8±50.8	***
Light (800<C≤3200)^1^	38	187.1±63.5	18	210.9±63.2	38	253.4±63.1	39	210.7±63.7	***
Moderate-to-Vigourous (C>3200)^1^	38	7.53±6.53	18	5.30±6.49	38	10.48±6.47	39	10.39±6.54	*

1 C = counts/minute.

2 *P<0.05, ***P<0.001, ns: non significant.

**Table 4:** Multiple regression models between RQ^1^, REE^2^, physical activity (dependant variables) and birth-weight, age, gender^3^ and H^2^/Z_200_(independent variables).

	Regressioncoefficient	S.E	p^4^	
*RQ*	*R^2^ = 0,08 p<0,01*			
Constant	0.750	0.019	***	
Sex	−0.160	0.086	ns	
Age (year)	0.054	0.086	ns	
Birth-weight (kg)	0.237	0.086	**	
***REE (kJ.d^−1^)***	***R^2^ = 0.27 p<0.001***			
Constant	1510.9	319.1	***	
Sex	−0.211	0.075	**	
Age (year)	0.456	0.076	***	
Birth-weight (kg)	0.200	0.076	**	
***REE (kJ.d^−1^)***	***R^2^ = 0.34 p<0.001***			
Constant	2982.4	237.7	***	
Sex	−0.103	0.074	ns	
Birth-weight (kg)	0.027	0.074	ns	
H^2^/Z_200_ 5	0.540	0.075	***	
***Total activity counts***(***counts.min^−1^.day^−^*** ^1^ ***)***	***R^2^ = 0.23 p<0.001***			
Constant	244.7	53.9	***	
Sex	−0.222	0.086	**	
Age (year)	0.427	0.086	***	
Birth-weight (kg)	−0.035	0.086	ns	

1 Respiratory quotient.

2 Resting energy expenditure.

3 girls = 1 and boys = 0.

4 **P<0.01, ***P<0.001, ns: non significant.

5 Residual H^2^/Z_200_ on age.

Girls were less active than boys (320±88 counts.min^−1^ and 350±88 counts.min^−1^, respectively, p* = *0.01). They also spent less time doing moderate-to-vigorous activities (p*<*0.001).

The mean birth-weight of each group of children was within normal ranges (Table 1) and was not statistically different between study groups.

Table 4 shows the results of four multiple regression models with RQ, REE or total activity counts as dependent variables and birth-weight, H^2^/Z_200_ age and gender as independent variables. These analyses reveal that RQ and REE are associated with birth-weight, the latter only when body composition (H^2^/Z_200_) is not accounted for. Birth-weight was not associated with total activity counts. However, in exploratory analysis, within the low birth-weights range (birth-weight≤2,5 kg, n = 10), birth-weight is negatively correlated with the time spent in minimal+sedentary activities (r_s_ = −0.7, *P = *0.04). In addition, within the high birth-weights range (birth-weight>4.2 kg, n = 11), birth-weight is negatively correlated with the time spent in moderate-to-vigorous activities (r_s_ = −0.8, p*<*0.001).

## Discussion

### Do Growth Retarded Children have Reduced fat Oxidation, Low Resting Energy Expenditure and/or Less Physical Activity Level?

In the present study, stunted children, whether or not overweight, had a substrate utilization similar to their non-stunted counterparts, whether or not overweight. Differences in children’s diet could challenge this result since RQ varies regarding fat and carbohydrates contents in diet. However, qualitative data show that stunted-overweight children differ from their counterparts only in the low diversity of their diet and their relatively low consumption of animal product. This suggests that growth retarded children do not have a reduced fat oxydation.

So far, very few studies have invastigated the occurrence of low rates of fat oxidation in growth retarded children. In one previous study, Guatemalan stunted children were reported to have no reduced fat oxidation [39]. However, in Brazil, stunted pre-adolescents had a reduced fat oxidation compared to normal stature pre-adolescents [17]. These conflicting results may have been caused by differences in research protocols. The RQs of Camerounian and Guatemalan children were calculated in empirical conditions. Conversely, in the Brazilian study, the stunted and non-stunted pre-adolescents were provided the same standardized diet during the 3-day measurement-period in order to limit the effect of differences in the diet of children on RQ. Beyond these methodological considerations, it must be emphasized that these studies considered populations living in a different environmental and cultural context. Bouchard [40] recently suggested that the predisposition to weight gain could be associated with several genes that could be combined in five, not mutually exclusive, genotypes resulting from several evolutionary pressures: “a thrifty genotype (low metabolic rate and insufficient thermogenesis), an hyperphagic genotype (poor regulation of appetite and satiety and propensity to overfeed), a sedens genotype (propensity to be couch potato and physically inactive), a low lipid oxidation genotype (propensity to be a low lipid oxidizer), and an adipogenesis genotype (ability to expand complement of adipocytes and high lipid storage capacity)”. The susceptibility for obesity, and hence the mechanisms involved in obesity, could therefore differ between populations depending on their genetic background and behavioral factors associated with their environment and culture.

In the present study, the low REE (kJ/d) found in stunted children (whether or not overweight) was associated with their body size, as reported previously [39], [41]. In fact, stunted children (as a group) have a higher REE per unit of body weight (kJ/d/kg of weight), as found in Brazilian stunted children [18]. It may result from a decreased lean mass in growth retarded children, which in turn induces a higher proportional contribution of the more metabolically active organs (brain and viscera) to total body size [42].

In line with previous findings [18], [43], stunted children (whether or not overweight) were found to be less active than their non-stunted (whether or not overweight) counterparts. This reduced level of physical activity may expose stunted children to a higher risk of in a context of nutrition transition [44]. Further studies should investigate the total energy expenditure of children, in particular the free living physical activity level component in relation to children’s diet. Low levels of physical activity in stunted children could result from chronic malnutrition or may be a behavioral adaptation to energy deficiency [20]. Reciprocally, the higher physical activity level of overweight *versus* stunted children could be associated with a higher energy availability. In addition, plumpy children may be more stimulated by their surroundings than malnourished children [45], [46]. The increase of physical activity level with age may be associated with modifications of the body size and/or composition, the improvement of the biomechanics of movement or scolarization (compulsory from 3 years old in Cameroon). In our study, girls were less active than boys and they spent more time doing sedentary activities, in accordance with previous findings [47]. Therefore, girls could be more at risk to develop overweight.

### Did Simultaneously Stunted and Overweight Children Suffer from Nutritional Deficiencies during Foetal Life?

The etiological model of the development of overweight in stunted children [15] suggests that early modifications in metabolic and behavioural pathways, leading to fat storage, are triggered by nutritional restrictions during foetal life and infancy. In the present study, birth-weight was used as an indicator of foetal development, as it was the most abundant data and the easiest parameter to obtain from mothers in Cameroon. In addition, low birth-weight is widely used as an indicator of fetal constraints and especially of the maternal nutritional deprivation during pregnancy. In this study, stunted and stunted-overweight children’s birth-weight was found within the normal range, suggesting that they did not experience foetal constraints. However, another recent study found that the effects of foetal constraints on birth-weight depends on the timing of disturbances, birth-weight being an inaccurate indicator of foetal growth restriction if it occurred before the third trimester [48]. Other foetal constraints, like potential factors of metabolic and growth disorders, and in particular maternal cardiometabolic diseases (diabetes, hypertension) and infections (HIV, malaria), will need to be considered in future studies.

On the other hand, it seems reasonable to suggest that post-natal nutritional restrictions may be among the factors of growth retardation in stunted-overweight children. Firstly, concerning breastfeeding, the majority of children, whatever their growth and nutritional status, are predominantly breastfed [11,unpublished data]. These results lead us to suspect that differences in growth and nutritional status in children may be associated with differences in weaning practices. Indeed, we found that stunted-overweight children were less likely to consume gruel enriched with sources of protein and infant cereals [unpublished data]. Furthermore, concerning post-weaning diet, our study reports that stunted-overweight children have a poorly diversified diet and consume less animal and dairy products.

In summary, these results suggest that during postnatal growth, stunted-overweight children were exposed to risk of deficiencies in proteins and/or micronutrients essential for growth [49]. Further studies measuring food intake quantitatively could help to corroborate these findings. Moreover, it would be interesting to work on the eating behaviour of stunted-overweight children since growth retarded children were found in a previous study to have an opportunistic eating behaviour [50].

### Relationships between Early Development and Fat Oxidation, Resting Energy Expenditure and Physical Activity

Relationships between birth-weight and energy utilisation, resting energy expenditure and physical activity were assessed to evaluate whether these parameters could be associated with foetal development. Birth-weight was indeed found to be predictive of substrates utilization *i.e.* increased birth-weight is associated to reduced lipid oxidation. We can therefore infer that lipid accumulation could be facilitated in children with a high birth-weight. This relationship may explain why high birth-weight was previously found to be a strong predictor of overweight in preschool children [51].

Variability of resting energy expenditure was strongly associated with the proxy of total body water (H^2^/Z_200_). The relationship between resting energy expenditure and birth-weight could therefore reflect that birth-weight is strongly predictive of lean mass, this later being greatly correlated with REE [52].

No relationship was found between birth-weight and overall activity (as measured by total activity counts). However, preliminary results suggest that low birth-weight children (<2.5 kg) tend to spend more time doing sedentary activities and that high birth-weight children (>4.2 kg) spend less time doing intense activities. These indicative results, to be validate in larger studies, suggest that decreased physical activity is associated with both low and high birth-weight, as recently found in adolescents and adults from Denmark, Finland, the Faroe Islands, Iceland, Norway and Sweden [53].

### Conclusion

In contrast with the etiological model considered here [15], neither impaired fat oxidation nor reduced resting energy expenditure were found to be *energy sparing mechanisms* in stunted children, whether or not these were overweight. However, in support of the model, stunted children, regardless of weight, have reduced levels of physical activity, making them more prone to develop overweight when supplied with a high energy diet. Stunted-overweight and stunted children have mean birth-weights within the normal range suggesting at least, an absence of nutritional deprivation during the third-trimester of maternal pregnancy. Their growth retardation could hence be attributed to postnatal factors like a diet low in sources of proteins and micronutrients, factors of optimal linear growth. Finally, concerning the relationship between foetal growth and metabolic and behavioural outputs during childhood, in this study birth-weight appeared to be weakly linearly associated with the utilization of substrates and with resting energy expenditure.

“The intergenerational effect hypothesis” is a complementary way to understand the coexistence of overweight and stunting in children [54], [15]. Early maternal biological changes could affect the child’s foetal and neonatal development (even if the mother lives in a healthier environment) which later predispose the child to higher risk to develop overweight. Several observations from our studies conducted in Cameroon suggest that the “intergenerational effect” should be explored to better understand the individual dual burden in children. In fact, Cameroon is experiencing a massive rural depopulation with a high proportion of malnourished persons migrating to towns where nutrition transition is ongoing and adult’s obesity and chronic disease are already a public health concern [55]–[56]. In the present study, 70% of mothers of stunted children were born in rural areas, while they formed only 30% of mother of non-stunted children. Mothers of stunted children are also the shortest in height (independantly of confounding factors) [11], which could be a consequence of early stresses (diet, disease…) affecting maternal growth [57]. These exploratory elements should stimulate future studies on the relevance of the intergenerational effect hypothesis for individual dual burden in children.
